# In Silico Analysis of the Effects of Omicron Spike Amino Acid Changes on the Interactions with Human Proteins

**DOI:** 10.3390/molecules27154827

**Published:** 2022-07-28

**Authors:** Nancy D’Arminio, Deborah Giordano, Bernardina Scafuri, Carmen Biancaniello, Mauro Petrillo, Angelo Facchiano, Anna Marabotti

**Affiliations:** 1 Department of Chemistry and Biology “A. Zambelli”, University of Salerno, Via Giovanni Paolo II, 132, 84084 Fisciano, Italy; ndarminio@unisa.it (N.D.); bscafuri@unisa.it (B.S.); 2 National Research Council, Institute of Food Science, 83100 Avellino, Italy; deborah.giordano@isa.cnr.it; 3 Department of Electrical Engineering and Information Technology, University of Naples “Federico II”, 80128 Naples, Italy; carmenbnl93@gmail.com; 4 Seidor Italy SRL, 21029 Milan, Italy; mauro.petrillo@seidor.com

**Keywords:** protein variants, spike, SARS-CoV-2, antibodies, protein–protein interactions

## Abstract

The SARS-CoV-2 variant Omicron is characterized, among others, by more than 30 amino acid changes occurring on the spike glycoprotein with respect to the original SARS-CoV-2 spike protein. We report a comprehensive analysis of the effects of the Omicron spike amino acid changes in the interaction with human antibodies, obtained by modeling them into selected publicly available resolved 3D structures of spike–antibody complexes and investigating the effects of these mutations at structural level. We predict that the interactions of Omicron spike with human antibodies can be either negatively or positively affected by amino acid changes, with a predicted total loss of interactions only in a few complexes. Moreover, our analysis applied also to the spike-ACE2 interaction predicts that these amino acid changes may increase Omicron transmissibility. Our approach can be used to better understand SARS-CoV-2 transmissibility, detectability, and epidemiology and represents a model to be adopted also in the case of other variants.

## 1. Introduction

A novel SARS-CoV-2 variant (B.1.1.529) was characterized and reported at the beginning of November 2021, initially in southern countries of Africa and subsequently in the rest of the world. On 26 November 2021, WHO classified variant B.1.1.529 as Variant of Concern (VOC) and named it “Omicron” [1].

Omicron carries more than 30 amino acid changes (including four deletions and one insertion), occurring on the spike glycoprotein, a key player protein in favoring the entrance of the virus into human cells by interacting with the human angiotensin-converting enzyme 2 (ACE2) receptor [2], and the final target of mRNA- and viral vector-based vaccines [3]. Consequently, at the time of its discovery, there was a common concern among scientists that both its transmissibility and antibody protection might be affected [4].

Immediately after the discovery of the SARS-CoV-2 virus in late 2019, the scientific community started a giant effort to characterize the structures of the proteins expressed by the viral genome in the shortest possible time. The first structure of the spike protein of SARS-CoV-2, obtained by cryo-electron microscopy (cryoEM) at a resolution of 3.46 Å [5], was released in the Protein Data Bank (PDB) [6]) in February 2020. Since then, more than 900 structures of SARS-CoV-2 spike protein in different conditions, obtained with different experimental approaches (mainly X-ray and cryoEM), have been made freely available (update: 31 May 2022). Many structures contain only the receptor-binding domain (RBD) of spike protein, which is essential for the recognition of the ACE2 human protein and subsequent membrane fusion and cell entry [7]. Many others contain the full-length spike protein, either monomeric or trimeric, carrying the RBD in different conformations: “up” form, suitable for ACE2 interaction, or “down” form. The first structure of a complex between the RBD spike protein (engineered to facilitate crystallization) and human ACE2 was released on 4 March 2020 [8]; a few days after, the first structure of the complex between a human antibody and the RBD of spike protein, was made available [9]. Since then, tens of structures of the complexes between the spike protein (either in its full form or fragments) and the human ACE2 receptor in different conditions, and hundreds of structures of the complexes of spike protein with different antibodies, have been solved and deposited in the PDB archive.

This impressive effort of the structural biology community has allowed the scientists to dissect the differences between SARS-CoV-2 spike protein and other spike proteins from known coronaviruses such as SARS-CoV and MERS-CoV (Middle East respiratory syndrome coronavirus). Moreover, it has been made possible to study the details of the interactions between the viral protein and the human cellular receptor and to understand the mechanism by which the viral particle is able to penetrate the cells, as well as the molecular reasons for its increased virulence. Furthermore, it has been made possible to assess the ability of antibodies developed against other coronaviruses to bind to the protein and block its recognition by ACE2 and to verify the mechanism of recognition of new antibodies (either developed by patients as a consequence of infection or following vaccines administration, or monoclonal antibodies) to infer their ability to protect people against reinfection.

This amount of structural information can be used as a starting point to predict the effect of the mutations developed by the different variants of the virus, thanks to computational methods that can help in predicting how a mutation can affect the interaction surface between spike protein and ACE2, or between spike protein and the antibodies known to bind and interact with it. For example, a recent study based on simulations of the interactions of the Omicron spike and human ACE2 found an increased number of electrostatic interactions and a lower binding free energy for the Omicron variant as compared to the reference spike protein [10]. It is noteworthy that a lot of in silico work is carried out within the coronavirus field. Just to cite different types of investigation, some studies focused on the mechanisms of interaction between spike protein and the main recognized human receptor, ACE2, by using bioinformatics and computational tools to compare sequences and dissect the molecular structures [11,12]. The sequence evolution among the variants has been studied by means of multiple sequence alignment and molecular modeling [13]. The role of SARS-CoV-2 human receptors in cancer pathologies has been investigated by mining bioinformatics resources on gene expression, disease networks, and functional pathways to explore comorbidity aspects [14]. Effects on the protein stability of the spike protein mutations observed in the circulating variants have been investigated by sequence alignments and predictions of differences in free energy due to the amino acid mutations [15]. Very recently, studies (appeared in literature after the submission of this manuscript and here added during the revision stage) investigated the Omicron variant interaction with a few antibodies active against the reference form of spike protein [16,17]. In this work, we selected several representative ensembles from the available structures of the complexes between spike protein and different antibodies as a starting point to model the amino acid changes affecting the spike protein in the Omicron variant of SARS-CoV-2, with the aim of understanding their impact on these interactions. In more detail, 157 complexes of spike protein with a single antibody were selected from representative 158 PDB ensembles, and amino acid replacements of the Omicron variants were simulated. The PDB complexes and the models generated in our study were compared by a detailed analysis to detect differences in the interactions occurring between spike protein and antibody. This work is part of a larger ongoing project to predict the impact of any possible mutation on the interactions that SARS-CoV-2 proteins may establish in the human body. Additionally, we performed an analysis to investigate the impact of each mutation on the interactions with ACE2 receptors. We hope that our predictions will help researchers better understand the impact that variants might have on both virus infectivity and the recognition by antibodies developed for other variants of the virus.

## 2. Results

### 2.1. Predicted Impact of Omicron Variant on Spike–Antibodies Interfaces

**Figure 1:**
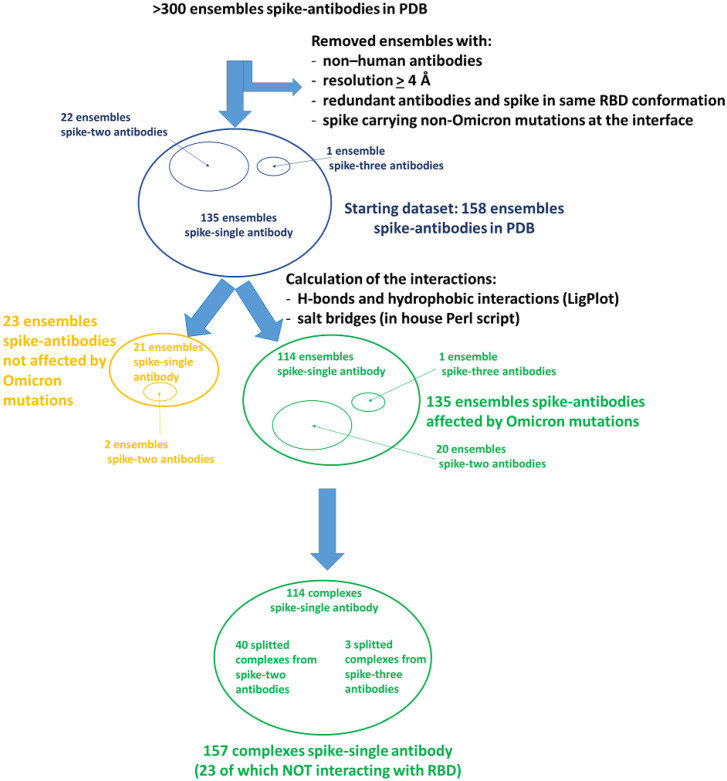
Flowchart of the procedure for the selection of the complexes to evaluate the interactions between spike and the antibodies.

We calculated the predicted impact of the amino acid replacements of the Omicron variant on the interactions present in the representative 158 PDB ensembles with spike protein and antibodies, selected as described in Methods. In total, 114 ensembles include only one antibody, 20 include two antibodies, and 1 includes three antibodies. The full list of the 158 selected ensembles is reported in Table S1. The amino acid replacements affected the original interface interactions in 135 out of 158 selected PDB ensembles. For the detailed analysis of the interactions, we split, among these 135 ensembles, those with two or three antibodies, obtaining 157 spike–single antibody complexes affected by amino acid replacements associated with Omicron spike protein. The flowchart of the whole procedure of selection is shown in Figure 1.

The detailed results are reported in Supplementary Table S2. Here, we summarize the most relevant findings.

In 134 out of these 157 single antibody–spike complexes, the antibody interacts with RBD; in the other 23 interact with other spike regions. In 68 out of these 157 complexes, the net effect of the amino acid replacements is the loss of interactions between the spike protein and the antibody (in 16 complexes, there is only a loss of interactions); in 60 out of these 68 complexes, the antibody interacts with RBD. In 70 out of the 157 complexes, the net effect of the amino acid replacements is the gain of interactions between the spike protein and the antibody (in 19 complexes, there is only a gain of interactions); in 59 out of these 70 complexes, the antibody interacts with RBD. Finally, in the last 19 out of these 157 complexes, the loss and gain of interactions are numerically equal; in 15 out of these 19 complexes, the antibody interacts with RBD.

**Figure 2:**
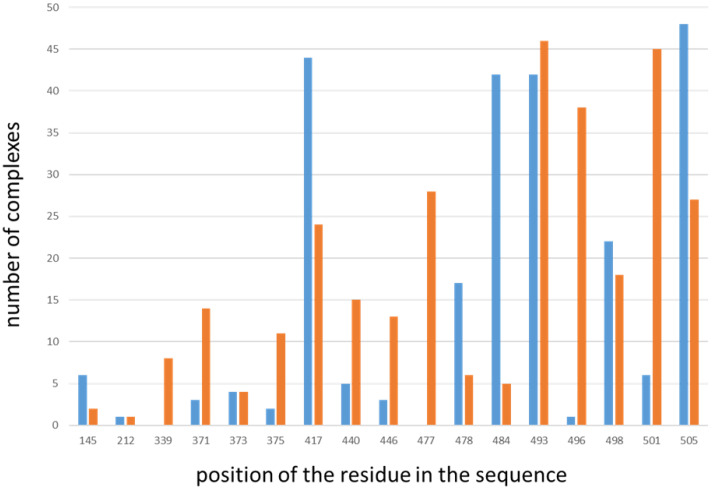
Number of complexes in which residues undergoing mutations in the Omicron variants cause the loss (blue bars) or gain (orange bars) of interactions. On the X axis is the position of the residue in the spike sequence; on the Y axis is the number of complexes in which the residue loses or gains interactions.

Of these 157 complexes, 127 have lost at least one interaction originally present in the PDB ensemble. Among them, 97 complexes have lost 50% or more of their original interactions (26 of which have lost all original interactions). Figure 2 shows, for those residues undergoing mutation in the Omicron variant that are involved in interactions with antibodies, how many complexes their replacement causes either a loss or a gain of interactions.

Only two amino acid replacements that cause the loss of some interactions (Y145D and L212I) do not affect residues belonging to the RBD. The amino acid replacements that cause the loss of interactions in the higher numbers of complexes are Y505H (in 48 complexes), K417N (in 44 complexes), E484A and Q493R (each in 42 complexes), whereas mutations L212I and G496S cause the loss of interactions in only one complex each (Figure 2, blue bars). However, it is worth noting that these residues affected by amino acids replacements in the Omicron variant are not equally present in all the selected ensembles, as shown in Table 1. Indeed, our dataset contains a total of 342 spike chains, but, for example, residue Y145D is visible only in 69 of them, while it is a missing residue in the other chains. Therefore, their relative frequency of occurrence in the dataset must be considered when looking at these data.

Looking at the type of interactions lost because of the amino acid replacements, we noticed that all the salt bridges originally present in the 157 complexes affected by these variations are lost after the amino acid replacements, whereas the other interactions (hydrogen bonds and/or hydrophobic interactions) are lost in a percentage variable from 10 to 100%.

Amino acid replacements associated with Omicron were also able to form new interactions in 128 out of 157 spike–single antibody complexes. Among them, in 99 complexes, 50% or more of the interactions after the amino acid replacement are new (34 of which have formed only new interactions). Y145D and L212I, the only two amino acid replacements not affecting the RBD, can cause the gain of interactions in a few complexes. New interactions are formed by Q493R in 46 complexes, by N501Y in 45 complexes, by G496S in 38 complexes, and by S477N in 28 complexes (Figure 2, orange bars). Additionally, in this case, it is necessary to take into account the different occurrences of these amino acids in the original spike chains present in our dataset.

Looking at the type of interactions lost because of the amino acid replacements, we noticed that only in one complex a new salt bridge was formed after amino acid replacements; the other new interactions formed in all complexes were both hydrogen bonds and hydrophobic interactions.

**Figure 3:**
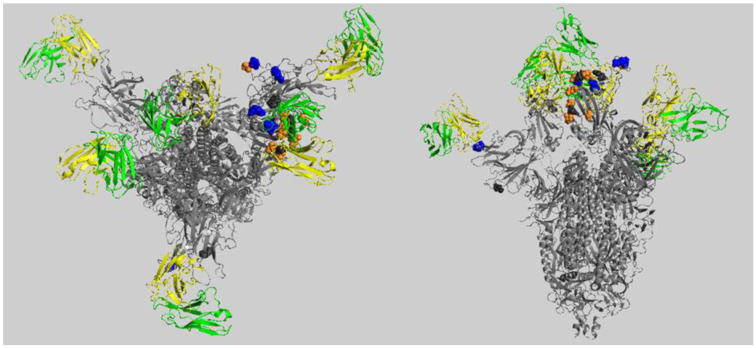
Position of the residues of spike protein involved in interactions negatively or positively affected by Omicron amino acid replacements in the higher number of complexes. In figure, we show as a representative complex the one in PDB file 7CWT, shown from the top of RBD (left panel) and from the side (right panel). The chains of spike are colored grey, the heavy chains of the antibodies visible in this complex are colored green, and the light chains are colored yellow. Residues mentioned in Figure 2 are shown in space-fill mode: in blue, those involved mainly in loss of interactions; in orange, those involved mainly in gain of interactions; in dark grey, those equally involved in loss and gain of interactions.

In Figure 3, we mapped the residues whose replacements cause the loss or the gain of interactions in a higher number of complexes onto the structure of spike protein in complex with hb27 and fc05 Fab (fragment antigen-binding) (PDB code: 7CWT), selected because it is one of the ensembles with the more complete structure of trimeric spike protein, containing all these residues. It is interesting to note that in the single spike chain, the residues causing loss of interactions (in blue) appear to be clustered together and spatially separated from those causing gain of interactions (in orange). The residues Tyr145 and Leu212 are far apart from RBD and from all the other residues and clearly interact with antibodies in a totally different part of the spike protein.

From our analysis, it appears that in six complexes, the antibodies lost all their interactions with spikes without gaining any new interaction. These antibodies are: CR3022, P5A-2G7, FC05, 2-51, DH1041, COVA1-16, and REGN10989 (in the PDB ensembles 7A5S, 7D03, 7D4G, 7L2C, 7LAA, and 7LQ7, respectively). In the other 21 cases where antibodies lost all their interactions with spike, we predicted new interactions forming after the amino acid replacement. In eleven cases, the net balance was still negative, but in seven cases, the number of new interactions was higher than the number of lost interactions, with a net positive balance. In two cases, finally, the number of new interactions formed equals the number of lost interactions.

The procedure adopted does not allow us to directly evaluate the effect of the insertions and of the deletions associated with the Omicron variant on the interactions between spike and the antibodies in our complexes. However, we evaluated how many times the residues of the spike affected by the deletions and the residue near the place of the insertion are involved in interactions with the antibodies in the 158 ensembles selected from PDB. Residues 70, 141, and 211 are not involved in any interaction in these ensembles; residue 69 is involved in interactions in one ensemble only, residue 142 in three, residue 143 in two, and residue 144 is involved in interactions in ten ensembles. Residue 214 is involved in interactions only in one ensemble. Thus, it might be deduced that these insertions and deletion do not cause a dramatic effect in the interaction of spikes with these antibodies.

### 2.2. Predicted Impact of Omicron Variant on Spike-ACE2 Interface

The interaction of the Omicron spike with ACE2 is object of interest in understanding how this variant can recognize its cellular receptor. Omicron spike glycoprotein shows seven amino acid replacements, not present in other variants, involving residues interacting with the ACE2 receptor. The net charge of the spike region interacting with ACE2 is increased (+3) by the amino acid changes related to the Omicron variant. As it is known that the counterpart on ACE2 is negatively charged [11,12], the mutations associated with the Omicron variant are expected to strengthen the stability of the interaction ACE2-spike by an electrostatic effect.

Our analysis suggests that the amino acid replacements occurring in the Omicron variant of spike protein may negatively affect the H-bonds that this protein can establish with the ACE2 receptor (Table 2).

**Figure 4:**
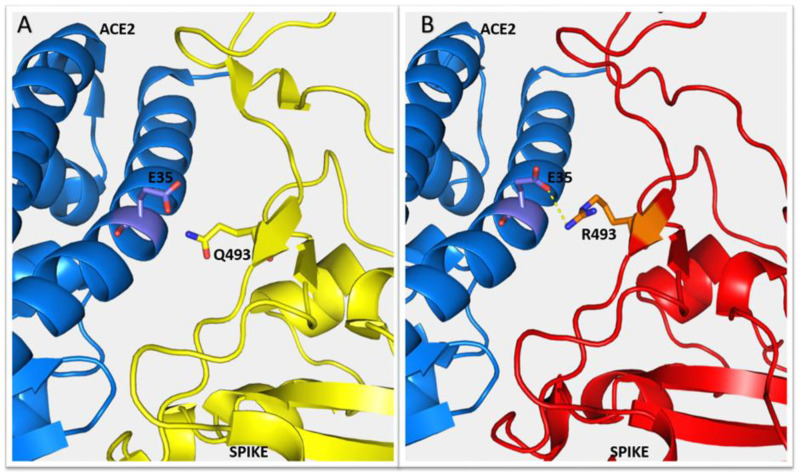
Possible salt bridge between ACE2 and spike protein because of amino acid replacements in Omicron variant. Panel (**A**): ACE2 backbone representation as blue ribbon, with the side chain of glutamate in position 35 in sticks, and original spike protein in yellow ribbon, with glutamine in position 493 in sticks. Panel (**B**): as for panel (**A**), with Omicron spike protein in red ribbon. In position 493, arginine replaces glutamine and the distance between the positively and negatively charged atoms of arginine and glutamate, respectively, is less than 4 Angstroms, suitable to form a salt-bridge (not possible with ACE2-original spike complex, due to the absence of the positive charge).

Concerning the analysis of salt bridge interactions, the mutation involving Lys417 (K417N) prevents the unique salt bridge observed for the spike protein form used for X-ray studies, i.e., between the spike Lys417 residue and ACE2 Asp30 [12]. However, we predict that new salt bridges may be formed with Omicron spike protein due to the amino acid replacements that introduce new charges. In particular, the suitable distance required for the formation of salt bridges is observed for the charged atoms of two residue couples, i.e., spike Arg493 with ACE2 Glu35 and spike His505 with ACE2 Glu37. Both spike residues are a consequence of substitutions (Q493R and Y505H). As an example, we show in Figure 4 the possible formation of the salt bridge between spike Arg493 with ACE2 Glu35. Therefore, the loss of the Lys417-Asp30 salt bridge may be counter-balanced or strengthened by new salt bridges formed because of the substitutions that occurred.

## 3. Discussion

The immediate sharing of sequences has facilitated the quick characterization of the novel Omicron SARS-CoV-2 variant. This is an evident demonstration of how immediate sharing of biological data is fundamental to quickly react to the appearance of potential novel variants of concern and, more in general, of biothreats.

Looking at the global effects of the amino acid replacements on the representative ensembles with antibodies extracted from the PDB archive, it appears an equilibrium between the loss of interactions and the gain of new interactions found in the complexes with the modeled Omicron spike mutations, with respect to those present in the former spike complexes. Therefore, we predict an overall modest impact of the Omicron variant on the spike recognition by these structurally characterized antibodies. The availability of higher resolution structures of spike–antibody ensembles will improve these predictions, as presently, only 43% of the ensembles we selected from PDB have a resolution better than 3 Å (see Supplementary Table S1), and many ensembles discarded by our dataset have a resolution worse than 4 Å. Moreover, in most spike structures, there are many missing residues that also include positions affected by amino acid replacements associated with the Omicron variant. Evidently, this aspect must be considered when interpreting the results and should be improved in future structures, as well as the codification of the different chains of the spike proteins and of the antibodies in the PDB file. Indeed, this lack of standardization causes the need for a very accurate manual check of the structures to be prepared for any automated analysis. It is obvious that, given their size and complexity, these structures create a great challenge to the structural biology community, and it is often difficult to relate them back to the PDB file standard, but in our opinion, some ad hoc rules for these structures would need to be introduced to improve their manageability by other scientists.

Concerning the interactions of Omicron spike variants with ACE2 receptor, our results are in good agreement with the recent predictive study [10] that proposed that the Omicron spike may increase the electrostatic interactions with ACE2 with respect to the original spike protein. However, we evidence that the formation of salt bridges is not certain, as the distance of charged atoms is lower than 4 Å, but the additional condition required in terms of distance of the charge centroids is not satisfied [18]. Therefore, it cannot be excluded that one or more of these changes (individually or in combination) can promote the increased Omicron variant transmissibility.

## 4. Materials and Methods

### 4.1. Definition of the Set of Omicron Amino Acid Changes

Omicron BA.1 spike glycoprotein shows 30 amino acids replacements with respect to the reference sequence of spike protein (UniProt code: P0DTC2): A67V, T95I, Y145D, L212I, G339D, S371L, S373P, S375F, K417N, N440K, G446S, S477N, T478K, E484A, Q493R, G496S, Q498R, N501Y, Y505H, T547K, D614G, H655Y, N679K, P681H, N764K, D796Y, N856K, Q954H, N969K, and L981F. Twelve of these amino acid replacements (underlined) are not reported in other VOCs. Those from residue 339 to 505 are within the RBD. Ten of these mutations (K417N, G446S, S477N, T478K, E484A, Q493R, G496S, Q498R, N501Y, and Y505H) involve amino acids interacting with the human ACE2 receptor. Additionally, the Omicron BA.1 variant shows four amino acid deletions: a double deletion H69-/V70-, typical of VOCs Alpha and Eta; a triple deletion G142-/V143-/Y144-, never reported in main VOCs but in other (sub)variants (A.2.5, P.3, A.2.5.2); a deletion N211-, never reported in main VOCs, but in other (sub)variants (AY.111; A.29; N.10); a deletion L141-, found only in a fraction of Omicron sequences (20%), and reported in (sub)variants (A.2.5, P.3, A.2.5.2). Furthermore, Omicron shows an insertion (ins214EPE) that to date is the first insertion found in a VOC of SARS-CoV-2.

### 4.2. Datasets Used for Structural Analyses

Among the ensembles of spike protein with different antibodies available in PDB, we manually selected 158 representative ensembles using the following criteria: i. presence of one or more non-redundant human antibodies or of a portion of them (usually Fab) in the ensemble; ii. completeness of the spike protein (we selected the trimeric form, whenever present; we selected the form with the lowest possible number of missing atoms and residues); iii. absence of mutations at the interfaces with antibodies (engineered spike proteins containing mutations far from interactions sites were included in our dataset); iv. RBD conformation (when complexes of the spike proteins and the same antibodies are available with RBD in different conformations, we kept all of them); v. resolution better than 4 Å. Twenty-two ensembles include two antibodies, and one ensemble includes three antibodies. The list of all selected spike–antibodies ensembles used as a starting point for this work is provided in Supplementary Table S1.

Among the structures of spike protein in complex with ACE2, we selected two representative structures, i.e., 6LZG [19] and 6M0J [20], being solved by X-ray crystallography, having resolution ≤2.50 Å, and having a wider coverage of the spike sequence.

### 4.3. Modeling and Analysis of the Effects of Mutations Associated with the Spike Protein in the Omicron Variant

For each spike–antibody ensemble selected from PDB, we first analyzed the interactions at the interfaces between the residues belonging to each chain of spike and each antibody chain, using an in-house R script to automatize the calculation of all possible combinations between each spike chain and each antibody chain. The interactions at the interface were analyzed by LigPlot+/DIMPLOT [21] and an in-house Perl script previously developed [22,23] to predict the presence of salt bridges following the criteria of Kumar and Nussinov [18] and to filter the interface interactions.

For the analysis of the Omicron variant, we modeled the missense mutations in the structure of spike protein, using a method already assessed in our previous studies about the effects of missense mutations on proteins [24,25]. Thus, for each ensemble selected from PDB, we introduced, one at a time, the amino acid substitutions belonging to the Omicron variant on the structure of spike proteins by means of the script mutate_model.py [26] associated to the well-known program for protein modeling MODELLER [27]. Then, we recalculated the interface interactions of the mutated ensembles as described above. Finally, we compared the interactions before and after the mutations, for each ensemble and globally, to predict the impact of each mutation on the interactions of the ensembles present in the PDB between spike and the antibodies, using in-house R scripts.

Models of spike protein complexed with ACE2 that include the amino acid replacements of the Omicron variant were obtained for both PDB complexes, i.e., 6LZG and 6M0J, by using MODELLER. For PDB complexes as well as modeled complexes, we then used again the programs LigPlot+/DIMPLOT and our in-house Perl script for spike–antibodies complexes to analyze the interactions at the interface for each antibody–spike complex.

Figures of molecular models were generated by the open source PyMOL Molecular Graphics System [28].

## 5. Conclusions

The impact of the Omicron variant on antibody recognition is the result of a complex balance of interactions. Our analysis suggests that, overall, this balance is affected but not dramatically perturbed by the amino acid replacements, insertions, and deletions carried out by the Omicron spike protein.

This kind of analysis could be extended to other SARS-CoV-2 proteins targeted by antibodies, such as the nucleocapsid protein, which is used for most immunoassay-based detection methods. Unfortunately, to date, only a few structures of antibodies–nucleocapsid protein complexes are available in PDB. We hope that in the future, more 3D data structures of these ensembles will become available. In this way, it will be possible to better assess the detectability of available immuno-based tests and devices with respect to the emerging of novel SARS-CoV-2 variants, a challenge of high concern for policymakers [29]. The scientific community should fill this gap as soon as possible: sharing data to facilitate COVID-19 testing and diagnostics is a must as relevant as understanding SARS-CoV-2 biology.

On the other hand, the developed approach also allows the evaluation of single mutations in their impact on the receptor and thus may be helpful in investigating the effects of new variants from the point of view of their transmissibility.

## Figures and Tables

**Table 1:** Summary of loss and gain of interactions in the spike–antibody complexes.

Residue Position in the Spike Sequence	Number of Complexes with Loss of Interactions Involving the Residue	Number of Complexes with Gain of Interactions Involving the Residue	Number of Spike Chains Including the Residue in the Starting Dataset (Total Number of Spike Chains in the Dataset: 342)
145	6	2	69
212	1	1	204
339	0	8	321
371	3	14	318
373	4	4	319
375	2	11	321
417	44	24	322
440	5	15	321
446	3	13	258
477	0	28	270
478	17	6	270
484	42	5	279
493	42	46	321
496	1	38	321
498	22	18	310
501	6	45	307
505	48	27	323

**Table 2:** List of H-bonds interactions at the interface of spike and human ACE2.

Residues Involved in H-Bonds in the Spike-ACE2 Interface		Reference Spike-ACE2 Complex		Omicron Spike-ACE2 Complex	
Spike Residue (in Parenthesis, the Replacement in Omicron Variant)	ACE2 Residue	6LZG	6M0J	6LZG mut	6M0J mut
LYS 417 (ASN)	ASP 30	+	+		
GLY 446 (SER)	GLN 42		+		+ (SER 446)
TYR 449	ASP 38	+	+		+
TYR 449	GLN 42	+	+		+
ALA 475	SER 19	+			
ASN 487	GLN 24	+	+		
ASN 487	TYR 83	+	+	+	+
GLN 493 (ARG)	GLU 35		+		
GLY 496 (SER)	LYS 353	+	+		
GLN 498 (ARG)	GLN 42	+			
THR 500	TYR 41	+	+	+	+
THR 500	ASP 355				+
THR 500	ARG 358				+
ASN 501 (TYR)	LYS 353				+ (TYR 500)
GLY 502	LYS 353	+	+	+	+
Total number of H-bonds		10	10	3	9

## Data Availability

Data are contained within the article or Supplementary Material.

## Supplementary Materials

The following supporting information can be downloaded at: https://www.mdpi.com/article/10.3390/molecules27154827/s1, Table S1: List of the 158 selected PDB ensembles of SARS-CoV-2 spike protein complexed with antibodies; Table S2: Detailed results of the analysis of spike–antibody complexes.

## References

[1] Classification of Omicron (B.1.1.529): SARS-CoV-2 Variant of Concern. https://www.who.int/news/item/26-11-2021-classification-of-Omicron-(b.1.1.529)-sars-cov-2-variant-of-concern.

[2] Scialo F., Daniele A., Amato F., Pastore L., Matera M.G., Cazzola M., Castaldo G., Bianco A. (2020). ACE2: The Major Cell Entry Receptor for SARS-CoV-2. Lung.

[3] Dai L., Gao G.F. (2021). Viral targets for vaccines against COVID-19. Nat. Rev. Immunol..

[4] Callaway E., Ledford H. (2021). How bad is Omicron? What scientists know so far. Nature.

[5] Wrapp D., Wang N., Corbett K.S., Goldsmith J.A., Hsieh C.L., Abiona O., Graham B.S., McLellan J.S. (2020). Cryo-EM structure of the 2019-nCoV spike in the prefusion conformation. Science.

[6] Berman H., Henrick K., Nakamura H. (2003). Announcing the worldwide Protein Data Bank. Nat. Struct. Biol..

[7] Xia X. (2021). Domains and Functions of Spike Protein in Sars-Cov-2 in the Context of Vaccine Design. Viruses.

[8] Shang J., Ye G., Shi K., Wan Y., Luo C., Aihara H., Geng Q., Auerbach A., Li F. (2020). Structural basis of receptor recognition by SARS-CoV-2. Nature.

[9] Yuan M., Wu N.C., Zhu X., Lee C.D., So R.T.Y., Lv H., Mok C.K.P., Wilson I.A. (2020). A highly conserved cryptic epitope in the receptor binding domains of SARS-CoV-2 and SARS-CoV. Science.

[10] Ortega J.T., Jastrzebska B., Rangel H.R. (2022). Omicron SARS-CoV-2 Variant Spike Protein Shows an Increased Affinity to the Human ACE2 Receptor: An In Silico Analysis. Pathogens.

[11] Xie Y., Karki C.B., Du D., Li H., Wang J., Sobitan A., Teng S., Tang Q., Li L. (2020). Spike proteins of SARS-CoV and SARS-CoV-2 utilize different mechanisms to bind with human ACE2. Front. Mol. Biosci..

[12] Giordano D., De Masi L., Argenio M.A., Facchiano A. (2021). Structural Dissection of Viral Spike-Protein Binding of SARS-CoV-2 and SARS-CoV-1 to the Human Angiotensin-Converting Enzyme 2 (ACE2) as Cellular Receptor. Biomedicines.

[13] Guérin P., Yahi N., Azzaz F., Chahinian H., Sabatier J.M., Fantini J. (2022). Structural Dynamics of the SARS-CoV-2 Spike Protein: A 2-Year Retrospective Analysis of SARS-CoV-2 Variants (from Alpha to Omicron) Reveals an Early Divergence between Conserved and Variable Epitopes. Molecules.

[14] Facchiano A., Facchiano F., Facchiano A. (2020). An investigation into the molecular basis of cancer comorbidities in coronavirus infection. FEBS Open Bio.

[15] Pascarella S., Ciccozzi M., Bianchi M., Benvenuto D., Giovanetti M., Cauda R., Cassone A. (2021). Shortening Epitopes to Survive: The Case of SARS-CoV-2 Lambda Variant. Biomolecules.

[16] Contractor D., Globisch C., Swaroop S., Jain A. (2022). Structural basis of Omicron immune evasion: A comparative computational study. Comput. Biol. Med..

[17] Xiong D., Zhao X., Luo S., Cong Y., Zhang J.Z.H., Duan L. (2022). Immune Escape Mechanisms of SARS-CoV-2 Delta and Omicron Variants against Two Monoclonal Antibodies That Received Emergency Use Authorization. J. Phys. Chem. Lett..

[18] Kumar S., Nussinov R. (1999). Salt bridge stability in monomeric proteins. J. Mol. Biol..

[19] Wang Q., Zhang Y., Wu L., Niu S., Song C., Zhang Z., Lu G., Qiao C., Hu Y., Yuen K.Y. (2020). Structural and Functional Basis of SARS-CoV-2 Entry by Using Human ACE2. Cell.

[20] Lan J., Ge J., Yu J., Shan S., Zhou H., Fan S., Zhang Q., Shi X., Wang Q., Zhang L. (2020). Structure of the SARS-CoV-2 spike receptor-binding domain bound to the ACE2 receptor. Nature.

[21] Laskowski R.A., Swindells M.B. (2011). LigPlot+: Multiple ligand-protein interaction diagrams for drug discovery. J. Chem. Inf. Model..

[22] Verdino A., D’Urso G., Tammone C., Scafuri B., Marabotti A. (2021). Analysis of the Structure-Function-Dynamics Relationships of GALT Enzyme and of Its Pathogenic Mutant p.Q188R: A Molecular Dynamics Simulation Study in Different Experimental Conditions. Molecules.

[23] Verdino A., D’Urso G., Tammone C., Scafuri B., Catapano L., Marabotti A. (2021). Simulation of the Interactions of Arginine with Wild-Type GALT Enzyme and the Classic Galactosemia-Related Mutant p.Q188R by a Computational Approach. Molecules.

[24] d’Acierno A., Scafuri B., Facchiano A., Marabotti A. (2018). The evolution of a Web resource: The Galactosemia Proteins Database 2.0. Hum. Mutat..

[25] Biancaniello C., D’Argenio A., Giordano D., Dotolo S., Scafuri B., Marabotti A., d’Acierno A., Tagliaferri R., Facchiano A. (2022). Investigating the Effects of Amino Acid Variations in Human Menin. Molecules.

[26] Modeller—Mutate_Model Script. https://salilab.org/modeller/wiki/Mutate%20model.

[27] Sali A., Blundell T.L. (1993). Comparative protein modelling by satisfaction of spatial restraints. J. Mol. Biol..

[28] PyMOL Molecular Graphics System. https://sourceforge.net/projects/pymol/.

[29] European Commission Technical Working Group on COVID-19 Diagnostic Tests. https://health.ec.europa.eu/health-security-and-infectious-diseases/crisis-management/covid-19-diagnostic-tests_en.

